# Gut Microbiota-Derived Metabolites to Regulate Intramuscular Fat Deposition in Pigs

**DOI:** 10.3390/microorganisms14020320

**Published:** 2026-01-29

**Authors:** Han Yuan, Lanlan Yi, Huijin Jia, Guangyao Song, Wenjie Cheng, Yuxiao Xie, Junhong Zhu, Sumei Zhao

**Affiliations:** 1 Yunnan Key Laboratory of Animal Nutrition and Feed Science, Yunnan Agricultural University, Kunming 650201, China; goldcaesium@outlook.com (H.Y.); yilanlan0217@163.com (L.Y.);; 2 College of Animal Science and Technology, Yunnan Agricultural University, Kunming 650201, China

**Keywords:** pig, intramuscular fat, gut microbiota, lipid metabolism, short-chain fatty acids, bile acids

## Abstract

Intramuscular fat (IMF) is a crucial determinant of pork quality, influencing tenderness, flavor, and consumer preferences, yet selective breeding has reduced its levels in modern pigs. This review explores the molecular and cellular mechanisms of IMF deposition, including progenitor cell differentiation via pathways like Wnt/β-catenin and PPARγ, and advances in non-invasive detection methods such as hyperspectral imaging and Raman spectroscopy. It highlights correlations and causal links between the gut microbiota composition and IMF, established through omics analyses, fecal microbiota transplantation, and germ-free models. Key microbial metabolites, including short-chain fatty acids (SCFAs) and bile acids, modulate lipid metabolism bidirectionally via signaling receptors like GPR43, FXR, and TGR5. Future research should integrate multi-omics and develop probiotics to enhance IMF efficiency for sustainable pork production.

## 1. Introduction

In 2024, global pork production reached 116.27 million tons, solidifying its position as the most consumed meat worldwide for the tenth consecutive year (after adjusting for the impact of African Swine Fever in 2020), surpassing chicken at 104.2 million tons and beef at 61.78 million tons. China remains the world’s largest producer and consumer of pork, with production projected to reach approximately 57.15 million tons in 2025, reinforcing its pivotal role in the global market [1].

Intramuscular fat (IMF) is a key biomarker of pork quality, typically assessed through marbling scores and sensory attributes, such as tenderness, flavor, and juiciness, alongside physical properties like drip loss, all of which collectively influence meat palatability. Over the past five decades, selective breeding in commercial pig breeds for a higher feed conversion efficiency and lean meat yield has reduced IMF levels to below 2%, potentially compromising consumer satisfaction. Consumer preferences have shifted from a focus on a high lean meat content toward a greater emphasis on the balance of flavor, nutrition, and cost. In Asian markets, particularly China, consumers are willing to pay a premium for pork with a superior flavor [2]. Consequently, the swine industry faces the challenge of establishing carcass standards that align with high-value market demands [3]. Improving pork quality, especially by enhancing IMF deposition efficiency, represents a critical objective in the field of animal grading science.

The gut microbiota, often referred to as the “second genome” of a species, exhibits a diversity, composition, and functionality influenced by factors such as diets, age, stress, and the environment [4]. These factors directly or indirectly affect the host metabolism, immune responses, and gut homeostasis [5,6]. The interaction between the gut microbiota and the host is commonly termed the “gut–X axis,” where X represents organs or tissues (e.g., liver, brain, or muscle) influenced by the microbiota [7]. Research has shown that the gut microbiota can modulate lipid metabolism in mice, pigs, and humans [8,9]. Given recent advances in understanding the role of the gut microbiota in lipid metabolism, exploring its impact on IMF deposition has emerged as a promising research direction. Based on a systematic literature search, this review synthesizes the molecular mechanisms of intramuscular fat (IMF) deposition in pigs and the role of gut microbiome-derived small molecules in its regulation via the “gut–muscle axis,” while outlining directions for future research.

## 2. Mechanisms and Detection Methods of IMF Deposition

### 2.1. Overview of IMF Deposition

Fat deposition in pigs reflects energy metabolic dynamics. When the net energy intake exceeds maintenance requirements, excess energy is partly converted into stored fat [10]. Notably, a substantial portion of the IMF may originate from the lipolysis of subcutaneous adipose tissue, which is then redistributed and stored within the muscle [11]. Studies in humans corroborate this, showing that insulin-sensitive, healthy athletes exhibit high IMF and low subcutaneous fat levels, likely due to the mobilization and redeposition of subcutaneous fat to high-energy-demand sites like muscle [12]. Additionally, IMF is derived from chylomicrons (triglycerides absorbed from the digestive system) and de novo lipogenesis in various intramuscular adipocytes [13].

### 2.2. Advances in IMF Quantitative Detection

An accurate IMF measurement is crucial for both research and livestock applications. Traditional methods, such as Soxhlet extraction and chemical identification, are destructive, labor-intensive, and time-consuming, making them unsuitable for live animal screening [14]. Most laboratories rely on gas chromatography and high-performance liquid chromatography (HPLC) for IMF analysis, which are also labor-intensive and time-consuming [15]. Emerging research focuses on leveraging large IMF datasets from traditional chemical methods to develop non-invasive techniques, such as ultrasound-based live IMF estimations and computer vision for large-scale automated IMF assessments [16].

Recent advancements include hyperspectral imaging (900–1700 nm) for predicting the IMF quality in pork loins, where texture features from hyperspectral images enable rapid and relatively accurate IMF content assessments [17]. Raman spectroscopy combined with chemometrics has successfully predicted the fatty acid composition in beef muscles, offering a faster and more accurate alternative to traditional methods [18]. In pork quality evaluations, models based on visual marbling scores and conventional meat quality traits have been developed to estimate IMF percentages, using SR (87.5%) and GBM (89%) models for rapid and accurate predictions in large sample sets [19]. Additionally, a three-stage deep learning model for ultrasound image analysis has improved the IMF prediction accuracy, achieving a mean absolute percentage error of 7.25% and an intraclass correlation coefficient of 0.905 with chemical measurements, outperforming existing technologies [20].

### 2.3. Cellular and Developmental Basis of IMF

The increase in IMF results from both the proliferation and hypertrophy of adipocytes within muscle tissue. During early growth stages in pigs, preadipocytes differentiate and proliferate into adipocytes, while during the fattening phase, the adipocyte volume increases, leading to greater fat deposition [21]. The IMF is regulated bidirectionally by lipogenesis and lipolysis, with net deposition occurring when the synthesis exceeds breakdown [22].

Three primary progenitor/stem cell types exist in the muscle microenvironment: muscle satellite cells (SCs), mesenchymal stem cells (MSCs), and fibro/adipogenic progenitors (FAPs) [23]. The SCs are muscle-resident stem cells located beneath the basal lamina of muscle fibers. They primarily differentiate into myocytes and are among the most abundant adult stem cell populations in muscle. Although SCs are not primary precursors for adipocytes, studies have observed lipid droplet accumulation in primary SCs under specific culture conditions, without initiating adipogenesis or expressing mature adipocyte marker genes. Under conditions such as increased insulin resistance, a reduced oxygen supply, or altered local metabolic environments, SCs exhibit increased fat accumulation [24,25].

The MSCs found systemically can differentiate into adipocytes, chondrocytes, and fibroblasts. During pig embryonic development, the differential regulation of transcription factors directs the MSC differentiation into myogenic, osteogenic, or adipogenic lineages, influencing the relative abundance of muscle, bone, and fat cells [26]. The Wnt/β-catenin signaling pathway inhibits an adipogenic differentiation of MSCs by suppressing key transcription factors, such as peroxisome proliferator-activated receptor gamma (PPARγ) and CCAAT/enhancer-binding protein alpha (C/EBPα), while promoting myogenesis and osteogenesis, making it a critical regulator of the muscle–fat balance [27]. Wnt proteins are glycoproteins that function as paracrine or autocrine signaling molecules. These proteins are released into the extracellular matrix by various cell types including fibro-adipogenic progenitors (FAPs), where they regulate cellular functions [28]. The β-catenin is degraded by enzymes when in an inactive state but translocates to the nucleus upon Wnt activation, where it regulates gene expression [29]. The upregulation of Wnt10b activates the Wnt/β-catenin pathway, leading to β-catenin interactions with lymphoid enhancer-binding factor 1 (Lef1) and runt-related transcription factor 2 (Runx2), increasing osteopontin (Opn) expression and enhancing the osteogenic potential of adipose-derived stem cells [30]. This process simultaneously suppresses PPARβ, C/EBPα, and fatty acid-binding protein 4 (FABP4) and eventually reduces adipogenesis [31,32,33]. Recent studies further highlight the inhibitory role of Wnt signaling in pig IMF deposition, with specific long non-coding RNAs (lncRNAs) and microRNAs (miRNAs) implicated in regulating IMF deposition [34].

Multiple signaling pathways modulate MSC differentiation. Bone morphogenetic proteins (BMPs) drive adipogenic differentiation through SMAD signaling, while zinc finger proteins (ZFPs) and sterol regulatory element-binding protein 1c (SREBP1c) enhance adipogenesis [35]. In contrast, the transforming growth factor beta (TGF-β)–SMAD3 signaling inhibits this process [27]. The SREBP1c is a key lipogenic transcription factor that is processed in the Golgi after binding to the endoplasmic reticulum and translocates to the nucleus to induce lipogenic gene expression [36].

FAPs are an independent mesenchymal cell population located in the skeletal muscle interstitium, capable of differentiating into fibroblasts and adipocytes [37]. The single-cell RNA sequencing of the longissimus dorsi muscle in Dahe and Dahe Black pigs identified FAPs as tenocytes involved in connective tissue and tendon function, committed preadipocytes that directly contribute to intramuscular fat (IMF), and interstitial cell populations that support intercellular communication [38]. This heterogeneity underscores the spatial and functional complexity of FAPs in IMF deposition, with a dual potential to differentiate into muscle or adipose lineages, predominantly occurring in perimysial and endomysial connective tissues [39,40]. While PPARγ is a master regulator of adipogenesis and typically induces the expression of downstream targets such as FABP4, the cited study [41] suggests that under specific conditions, the absence of FABP4 may be associated with upregulated PPARγ expression and enhanced adipocyte differentiation. This indicates a potential complex regulatory relationship where FABP4 might exert context-dependent effects on PPARγ activity and IMF deposition, possibly through lipid-mediated signaling or feedback mechanisms.

Another pathway in preadipocytes involves the inhibition of nucleotide biosynthesis, leading to altered mitochondrial cristae structures and the reduced expression of oxidative phosphorylation (OXPHOS) proteins [42]. Restoring PPARγ expression under these conditions reinstates OXPHOS protein levels [43]. Nucleotide biosynthesis inhibition also reprograms mitochondrial metabolism, reducing tricarboxylic acid cycle intermediates and increasing fatty acid oxidation (FAO) [44]. Suppressing FAO restores PPARγ, mitochondrial proteins, and adipogenic markers, indicating a positive feedback loop between mitochondrial catabolic intermediates and PPARγ in regulating adipogenesis [17].

The progenitor/stem cell populations contribute to the connective and adipose tissues in meat, with defined pathways guiding differentiation into all major cell types found in muscle tissue [18]. In summary, IMF deposition in pigs is a complex physiological and biochemical process without a single, linear regulatory node, necessitating further research into the crosstalk among metabolic pathways [19].

The crosstalk among these key signaling pathways forms a dynamic and interconnected regulatory network, rather than isolated linear cascades, which collectively determines the adipogenic fate of muscle-resident progenitor/stem cells and precisely modulates intramuscular fat (IMF) deposition. The Wnt/β-catenin pathway acts as a master inhibitory switch that maintains myogenic or stromal potential in MSCs and FAPs by constitutively suppressing core adipogenic transcription factors such as PPARγ and C/EBPα. When pro-adipogenic signals prevail, the BMP-SMAD and SREBP1c pathways are activated. These pathways not only directly promote adipogenic gene programs but may also downregulate or antagonize Wnt signaling, thereby relieving the repression on PPARγ. PPARγ resides at the hub of this network, integrating inputs from upstream pathways, including Wnt, BMP, and SREBP1c. Once sufficiently activated, PPARγ not only drives terminal adipogenesis but also engages in a positive feedback loop with mitochondrial metabolism (e.g., OXPHOS and FAO), thereby stabilizing and amplifying the adipogenic signal through metabolic reprogramming. Concurrently, inhibitory pathways such as TGF-β/SMAD3 provide a counterbalance to prevent excessive lipid accumulation. Thus, the net IMF deposition is ultimately determined by the dynamic equilibrium between pro-adipogenic (BMP, SREBP1c, and PPARγ) and anti-adipogenic (Wnt and TGF-β) signaling modules within this network, influenced by the developmental stage and local microenvironmental cues (e.g., nutritional and endocrine status). Unraveling the cross-regulatory interactions, especially the integrated control at key nodes like PPARγ, is essential for a holistic understanding of the complexity underlying IMF deposition.

## 3. Correlation and Causality Between IMF and Gut Microbiota

Research on the IMF and gut microbiota focuses on two main aspects: correlation and causality. We summarize the recent progress in understanding how body fat phenotypes relate to the gut microbiota composition. Studies indicate differences in the gut microbiota composition between obese and normal-weight individuals. Consistent with human studies, the data on Guizhou minipigs have shown a higher Firmicutes-to-Bacteroidetes ratio in obese castrated males and obese females than in non-obese females. Comparisons between obese Meishan pigs and lean Yorkshire pigs revealed that Meishan pigs exhibit higher dietary fiber digestibility, an increased abundance of polysaccharide-degrading bacteria (e.g., Bacteroides, Spirochaeta, and Prevotella), SCFAs, hydrogenotrophic microbes (e.g., Methanobrevibacter and Blautia), and methane and acetate production pathways [20]. These findings highlight the relationship between the gut microbiota and host metabolic state, providing evidence from animal models [45].

The metagenomic sequencing of colon microbiota in high-fat and low-fat Jinhua pigs revealed distinct microbial compositions [46]. High-fat pigs had higher relative abundances of Firmicutes and Tenericutes, while low-fat pigs showed an enrichment of Ruminococcus, Faecalibacterium, and Vibrio. Notably, variations in methanogenic archaea and butyrate-producing bacteria correlated with host fat metabolism phenotypes. Molecular analyses further indicated a higher expression of fatty acid synthase and PPARγ in the abdominal adipose tissue of high-fat pigs, providing evidence of microbiota interactions with the host lipid metabolism [47]. In obese mice, the Bacteroidetes abundance decreased by 50%, while Firmicutes increased proportionally, independent of the genetic background, suggesting that obesity alters the gut microbial diversity and composition [48]. Similarly, in type 2 diabetes and obese patients, the Firmicutes abundance increased, and Bacteroidetes decreased relative to healthy individuals [49,50]. It is important to note, however, that the Firmicutes-to-Bacteroidetes (F/B) ratio as a biomarker for obesity is a subject of ongoing debate, with findings often inconsistent across studies. Studies have also found increased microbial diversity in obese individuals, with the F/B ratio rising with BMIs up to 33 but declining thereafter [51]. These observations highlight that the association between the F/B ratio and obesity is complex and non-linear and is significantly influenced by factors such as diet, genetics, and cohort characteristics. An analysis of the gut microbiota in twins with differing body fat levels showed a lower microbial diversity in obese individuals [52]. Collectively, this body of work provides evidence for a link between the gut microbiota and host lipid metabolism, although caution is warranted when interpreting single metrics like the F/B ratio as universal indicators.

While omics studies demonstrate correlations between the gut microbiota, their metabolites, and IMF deposition, they lack a causal inference. However, germ-free animal models and fecal microbiota transplantation (FMT) experiments provide causality for the interaction of the gut microbiota with the host lipid metabolism [53]. Hence, emerging techniques like FMT and single-strain colonization are increasingly vital in studying fatty acid compositions and fat deposition regulation. High-throughput sequencing demonstrated that lean Yorkshire (RP) pigs had a higher Firmicutes-to-Bacteroidetes ratio and distinct genus-level differences compared to Rongchang pigs. The FMT experiments showed that transplanting microbiota from RP pigs into germ-free mice replicated the donor phenotypes, where the RP mice exhibited increased body fat, higher slow-twitch fiber proportions, reduced fast-twitch IIb fiber proportions, smaller fiber diameters, and enhanced adipogenic activity in the gastrocnemius muscle of germ-free mice. The microbiota of the recipient mice resembled that of their donors. All these findings provide evidence for the role of the gut microbiota in the regulation of host muscle development and lipid metabolism [54].

A study has shown that Shaziling pigs had a higher probiotic abundance compared with Yorkshire pigs, with *Lactobacillus johnsonii* identified as a key species influencing lipid metabolism. *Lactobacillus johnsonii* is expected to affect the host metabolism by promoting lipid absorption and transport. Transplantation experiments with *L. johnsonii* in DLY pigs resulted in elevated saturated fatty acid (SFA) contents in muscle tissue that was linked to a higher expression of genes such as DGAT1, DGAT2, CD36, and PPARγ, which are all related to lipid metabolism. These findings provide critical insights into the molecular mechanisms by which specific gut microbes regulate the host lipid metabolism [54].

A comparative analysis of the gut microbiota in Laiwu and DLY pigs revealed that transplanting Laiwu pig microbiota into DLY piglets increased the intramuscular fat (IMF) content in the longissimus dorsi muscle. Integrated metagenomic and metabolomic analyses identified four key functional bacterial species—*Bacteroides uniformis*, *Sphaerochaeta globosa*, *Hydrogenoanaerobacterium saccharovorans*, and *Pyramidobacter piscolens*—that likely influence IMF deposition by modulating host lipid metabolism pathways. These species upregulate the expression of fatty acid desaturase 1, FABP4, and fatty acid synthase while inhibiting adenosine monophosphate-activated protein kinase activity, offering a theoretical basis for improving meat quality through microbial interventions [55].

The FMT experiments further elucidated the mechanisms of the gut microbiota in regulating IMF deposition. Transplanting microbiota from Jinhua and Landrace pigs into mice revealed that recipients of the Jinhua pig microbiota exhibited an increased IMF content. Mechanistic studies indicated the upregulation of lipoprotein lipase (LPL) expression in gastrocnemius muscle, the downregulation of angiopoietin-like protein 4 (ANGPTL4) expression, and reduced concentrations of short-chain fatty acid (SCFA) levels in the colon. The SCFAs are known to promote ANGPTL4 transcription and secretion, which inhibits LPL. The Jinhua pig-derived microbiota likely reduces the abundance of bacteria that synthesis SCFAs. The low concentration of SCFAs weakens ANGPTL4-mediated LPL inhibition and ultimately promotes fat deposition in muscle tissue [56]. Together, these findings illustrate that the SCFA-mediated regulation of IMF deposition is context-dependent, varying according to the microbial composition, tissue specificity, and SCFA concentration.

## 4. Mechanisms of Microbial Metabolites for Regulating IMF Deposition

The gut microbiome likely influences IMF deposition through small-molecule metabolites acting as signaling molecules or energy substrates. This section reviews recent research on how microbial metabolites interact with host molecular networks to regulate adipogenesis, lipid metabolism, and muscle–lipid crosstalk.

### 4.1. Short-Chain Fatty Acids

The SCFAs, including acetate, propionate, and butyrate, are produced by the gut microbiota through the anaerobic fermentation of dietary fiber. The SCFAs play a pivotal role in energy supply and lipid metabolism [57]. Different microbial taxa produce distinct SCFA profiles. Bacteroidetes primarily generate acetate and propionate, while Firmicutes predominantly produce butyrate [58]. The SCFAs serve as substrates for lipid synthesis. Studies demonstrate that acetate and butyrate are converted into acetyl-CoA, a primary substrate for de novo lipogenesis in rat colonic epithelial cells [59]. Additionally, microbial acetate acts as a precursor for palmitic and stearic acid synthesis, regulating hepatic fatty acid metabolism [60].

Beyond their role as substrates, SCFAs function as signaling molecules in lipid metabolism [61]. Their impact on IMF is complex and bidirectional. On one hand, SCFAs promote lipid synthesis by providing substrates and activating lipogenic genes [62]. On the other hand, they enhance energy expenditure through pathways like PPARγ and AMPK, potentially reducing fat accumulation [63]. Consequently, the net effect of SCFAs on IMF deposition depends on specific conditions and metabolic states [64]. G-protein-coupled receptor 43 (GPR43) mitigates diet-induced obesity by increasing glucagon-like peptide-1 (GLP-1) secretion. Acetate induces anti-lipolytic activity in white adipose tissue via GPR43, improving glucose and lipid metabolism [65]. Propionate and butyrate activate PPARγ, promoting energy expenditure, reducing body fat, and lowering hepatic triglyceride deposition [66]. Butyrate activates brown adipose tissue via the gut–brain axis, enhancing fatty acid oxidation and ameliorating diet-induced obesity and insulin resistance [67]. Additionally, butyrate regulates energy expenditure by activating AMPK and inducing peroxisome proliferator-activated receptor-γ coactivator-1α, promoting white adipose tissue browning and reducing the adipocyte size [68]. In pigs, butyrate enhances adipogenesis in stromal vascular cells, likely by upregulating PPARγ, C/EBPα/β, and SREBP-1c expression, increasing lipid accumulation and potentially IMF deposition through preadipocyte differentiation [69].

In short, SCFAs are critical for maintaining lipid metabolism and energy homeostasis. Acetate supplementation reduces body weight and improves glucose tolerance in obese and diabetic rats [70]. Butyrate inhibits obesity and enhances thermogenesis in mice [71,72]. Propionate and butyrate improve glucose homeostasis [73], while propionate precursors and acetate increase plasma peptide YY and GLP-1 levels, reducing the energy intake and body weight gain [43,74].

Additionally, SCFAs and bile acids produced by the gut microbiota suppress PPARγ signaling to inhibit adipogenesis. Folic acid supplementation reduces the Firmicutes-to-Bacteroidetes ratio, increasing Alistipes, Oscillospira, Ruminococcus, Clostridium, Dehalobacterium, and Parabacteroides abundances. Moreover, folic acid elevates acetate and propionate levels and shows a negative correlation with adipocyte proliferation and differentiation genes such as C/EBPα and PPARγ, ultimately inhibiting fat accumulation [75,76]. The FMT from folic acid-treated donors further reduces fat deposition by downregulating PPARγ, C/EBPα, and FABP4 expression [77]. Gut microbiota directly or indirectly modulate adipocyte differentiation via PPARγ signaling, but their complex interactions with other microbes and metabolites require further investigation [44].

Clostridium butyricum produces butyrate, which suppresses Wnt/β-catenin signaling and alters the gut microbiota composition by reducing pathogenic and bile acid-metabolizing bacteria while increasing beneficial SCFA-producing bacteria. This activates GPR43 and GPR109A, inhibiting tumor cell proliferation [78,79]. Butyrate also stabilizes the gut mucosal barrier via the M2 macrophage–Wnt ligand–ERK1/2-MUC2/SPDEF pathway [80]. The subset of intestinal macrophages known as CD206+ macrophages secretes Wnt ligands to maintain mesenchymal cell niches critical for Paneth cell differentiation. Lactobacillus colonization or the transfer of CD206+ macrophage promotes Paneth cell differentiation and reduces colitis development [81]. Butyrate from Lactobacillus rhamnosus GG expands regulatory T cell pools, increases Wnt10b production by CD8+ T cells, and stimulates bone formation by promoting MSC differentiation into osteoblasts while inhibiting adipogenesis [82,83].

Despite their simplicity, SCFAs play a critical role in IMF deposition with dual regulatory effects, capable of both promoting and inhibiting fat accumulation. Identifying optimal bacterial combinations and SCFA concentrations is essential to harness their potential for enhancing IMF deposition [84].

### 4.2. Bile Acids (BAs)

Primary bile acids, synthesized from cholesterol in the liver and conjugated with taurine or glycine, are stored in the gallbladder and are secreted into the duodenum to facilitate lipid metabolism [85]. Conjugated bile acids are deconjugated by gut bacteria and bile salt hydrolases to form free bile acids, which are further metabolized into secondary bile acids via bacterial dehydrogenation and dehydroxylation in the colon [86]. The microbial metabolism increases bile acid hydrophobicity, promoting fecal excretion. Beyond their role in lipid digestion, bile acids act as signaling molecules by binding to nuclear receptor farnesoid X receptor (FXR) and G-protein-coupled bile acid receptor 5 (TGR5), hence regulating host metabolism. Microbial metabolism diversifies bile acids, with secondary bile acids constituting approximately 5% of the total pool, acting as agonists or antagonists depending on receptor affinity [87,88].

Primary bile acids, such as cholic acid (CA) and chenodeoxycholic acid (CDCA), and secondary bile acids, such as lithocholic acid (LCA) and deoxycholic acid (DCA), are FXR agonists. While CDCA can be converted to ursodeoxycholic acid, which is an FXR antagonist [89], the FXR plays a key role in triglyceride transport, synthesis, and utilization [90]. Microbial bile acid metabolism thus modulates animal lipid metabolism via FXR interactions. Microbiota-mediated weight gain and hepatic steatosis are FXR-dependent, as shown in a study where FXR-knockout mice were fed a high-fat diet [91]. Transplanting microbiota from FXR-knockout mice into germ-free mice reduces body fat, indicating that the FXR influences fat deposition by altering the gut microbiota structure [92].

**Figure 1:**
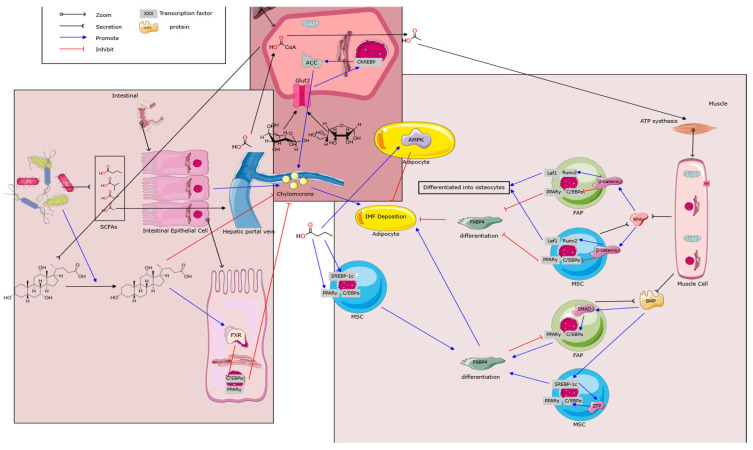
A schematic overview of the metabolic crosstalk between the intestine, liver, and skeletal muscle in regulating intramuscular fat (IMF) deposition. Dietary nutrients are absorbed by intestinal epithelial cells, assembled into chylomicrons, and transported via the hepatic portal vein to the liver, where lipid and glucose metabolism are regulated through FXR, AMPK, and SREBP-1c signaling. Short-chain fatty acids (SCFAs) derived from gut fermentation further modulate hepatic energy homeostasis. In muscle, mesenchymal stem cells (MSCs) differentiate into adipocytes under the control of PPARγ, C/EBPα, Wnt/β-catenin, and BMP pathways, promoting IMF accumulation. This integrative network highlights the intestine–liver–muscle axis regulation of lipid synthesis, transport, and storage. ATP: Adenosine Triphosphate. ACC: Acetyl-CoA Carboxylase. IMF: Intramuscular Fat. Wnt: Wingless-Type (signaling pathway). ChREBP: Carbohydrate Response Element Binding Protein. FAP: Fibro/Adipogenic Progenitor. Lef1: Lymphoid Enhancer-Binding Factor 1. Runx2: Runt-Related Transcription Factor 2. PPARγ: Peroxisome Proliferator-Activated Receptor Gamma. C/EBPα: CCAAT/Enhancer-Binding Protein Alpha. SMAD: Mothers Against Decapentaplegic Homolog. MSC: Mesenchymal Stem Cell. BMP: Bone Morphogenetic Protein. FABP4: Fatty Acid-Binding Protein 4. SREBP-1c: Sterol Regulatory Element-Binding Protein 1c. ZFP: Zinc Finger Protein. SCFAs: Short-Chain Fatty Acids. Glut2: Glucose Transporter 2. CoA: Coenzyme A. FXR: Farnesoid X Receptor. AMPK: AMP-Activated Protein Kinase.

Bile acids also regulate host lipid metabolism via TGR5. The TGR5 activation in skeletal muscle and brown adipose tissue promotes energy expenditure, improving the host metabolism and reducing obesity. The TGR5 activation also enhances the GLP-1 release, mitigating obesity and insulin resistance [93]. Microbial-derived LCA and DCA act as TGR5 agonists, modulating lipid metabolism [94]. Secondary bile acids suppress PPARγ and SREBP1c expression via FXR and TGR5 signaling, ultimately reducing adipogenesis [95,96]. This indicates a bidirectional relationship where the microbial metabolism modulates the lipid metabolism via FXR interactions. Related mechanisms are shown in Figure 1.

## 5. Summary and Future Perspectives

Enhancing the efficiency of IMF deposition is a critical challenge in animal science. However, current research faces several limitations, including the molecular mechanisms of microbiota–host interactions, which are highly complex and challenging to fully elucidate, particularly the synergistic or antagonistic effects of microbial communities on IMF. Most studies rely on mouse models or human data, and their applicability to pigs requires further validation. Economically, increasing IMF while reducing backfat thickness presents a seemingly contradictory goal, demanding precise targeted regulation. The lack of standardized microbiome data (e.g., metagenomics, transcriptomics, and metabolomics) hinders cross-study comparability.

Future research should focus on integrating multi-omics analyses and leveraging technologies like spatial transcriptomics to systematically dissect microbiota–host interaction networks and identify key regulatory nodes. Screening bacterial strains with high IMF may promote efficiency to develop probiotics or synbiotics for targeted microbiota interventions. Standardizing non-destructive detection technologies, e.g., hyperspectral imaging or Raman spectroscopy, can improve live animal IMF screening efficiency, and studies should draw on human metabolic disease research to balance IMF deposition with health risks in pigs. These advancements will likely transition the swine industry from empirical breeding to precision regulation, meeting consumer demands for high-quality pork while promoting sustainable industry development.

## Data Availability

No new data were created or analyzed in this study. Data sharing is not applicable to this article.

## Abbreviations

The following abbreviations are used in this manuscript:

IMF	Intramuscular Fat
GBM	Gradient Boosting Machine
MAPE	Mean Absolute Percentage Error
ICC	Intraclass Correlation Coefficient
SCs	Satellite Cells
MSCs	Mesenchymal Stem Cells
FAPs	Fibro/Adipogenic Progenitors
BAT	Brown Adipose Tissue
PPARγ	Peroxisome Proliferator-Activated Receptor Gamma
C/EBPα	CCAAT/Enhancer Binding Protein Alpha
FABP4	Fatty Acid Binding Protein 4
BMPs	Bone Morphogenetic Proteins
SMAD	Small Mothers Against Decapentaplegic
SREBP1c	Sterol Regulatory Element-Binding Protein 1c
TGF-β	Transforming Growth Factor Beta
SR	Stepwise Regression
EIMFS	Enhanced Intramuscular Fat Score
GC	Gas Chromatography
HPLC	High-Performance Liquid Chromatography
SCFAs	Short-Chain Fatty Acids
GPR43	G Protein-Coupled Receptor 43
GLP-1	Glucagon-Like Peptide 1
AMPK	AMP-Activated Protein Kinase
PGC-1α	Peroxisome Proliferator-Activated Receptor Gamma Coactivator 1 Alpha
FXR	Farnesoid X Receptor
TGR5	Takeda G Protein-Coupled Receptor 5
TG	Triglyceride
UDCA	Ursodeoxycholic Acid
LCA	Lithocholic Acid
DCA	Deoxycholic Acid
CA	Cholic Acid
CDCA	Chenodeoxycholic Acid
BSH	Bile Salt Hydrolase
